# Responsive Membrane Antenna Enables Adaptive Biohybrid Interface for Photosynthesis Promotion, Stress Reporting and Modulation

**DOI:** 10.1002/advs.77205

**Published:** 2026-08-15

**Authors:** Bing Liu, Gongmeiyue Su, Jingyi Shi, Hang Yao, Yuchun Han, Zhao Li, Chengcheng Zhou

**Affiliations:** ^1^ Jiangsu Provincial Key Laboratory of Green & Functional Materials and Environmental Chemistry School of Chemistry and Materials Yangzhou University Yangzhou People's Republic of China; ^2^ School of Medical Science and Engineering School of Materials Science and Engineering Beijing Institute of Technology Beijing People's Republic of China; ^3^ CAS Key Laboratory of Colloid Interface and Chemical Thermodynamics Institute of Chemistry Chinese Academy of Sciences Beijing People's Republic of China; ^4^ University of Chinese Academy of Sciences Beijing People's Republic of China

**Keywords:** aggregation‐induced emission, membrane antenna, photosynthesis, self‐assembly, stress tolerance

## Abstract

Biohybrids promise to endow photosynthetic organisms with new or enhanced functions, yet most operate monofunctionally, targeting either photosynthesis enhancement or stress tolerance, and cannot report environmental changes. Here, we develop an environmentally responsive membrane antenna, TPyB, featuring an aggregation‐induced emission light‐harvesting module with an H_2_O_2_‐cleavable site. Assembled onto the plasma membrane of green microalgae, TPyB creates a biohybrid with a responsive interface for photosynthesis promotion, stress reporting, and modulation. Under normal conditions, TPyB acts as a spectral converter that reconfigures incident sunlight to augment photosynthesis and biomass. Upon stress onset, rising intracellular H_2_O_2_ triggers the cleavage of TPyB, producing dual outputs: a distinct fluorescence color change (red to yellow) for stress reporting, and direct H_2_O_2_ quenching to alleviate oxidative damage. This trait enables the biohybrid to conditionally shift from light optimization to stress management, achieving faster recovery and enhanced tolerance. Thus, by assembling a responsive membrane‐anchored antenna with living organisms, this work establishes a bioaugmentation paradigm for advanced biohybrids capable of environmental sensing and adaptive regulation.

## Graphical abstract

An environmentally responsive antenna assembled onto the algal cell membrane creates a biohybrid with a responsive interface that promotes photosynthesis under normal conditions and shifts to H_2_O_2_‐triggered stress reporting and modulation upon stress onset, boosting environmental sensing and adaptive response.

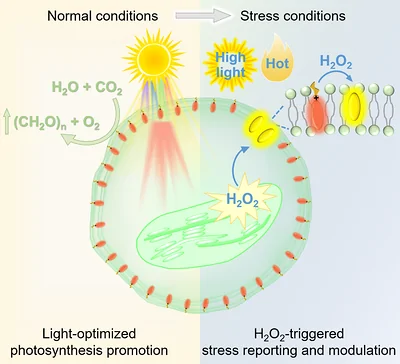

## 1 Introduction

Photosynthesis captures sunlight to convert CO_2_ and water into biomass, shaping Earth's climate and sustaining global food and energy supply [1, 2, 3]. Yet its solar‐to‐biomass efficiency is merely 1%–3%, far below the theoretical limit of ∼12% [4, 5]. This gap stems from an evolutionary trade‐off in photosynthetic organisms that prioritizes survival over maximal energy conversion [6, 7]. One manifestation is the narrow photosynthetically active radiation (PAR) range: the photosynthetic apparatus primarily absorbs blue and red light, while discarding disruptive ultraviolet (UV), less energetic far‐red light, and most green light, leaving nearly half of the incident sunlight untapped [8]. Even within PAR, energy conversion is suboptimal because blue light carries more energy than photochemistry can effectively harness [9]. To avoid photodamage, photosynthetic organisms have evolved non‐photochemical quenching (NPQ) [10], which dissipates excess energy as heat but compromises photosynthetic efficiency. These inherent inefficiencies are exacerbated by environmental fluctuations, particularly high light (HL) and high temperature (HT) [11, 12, 13, 14], both intensifying with climate change. Such stressors disrupt cellular homeostasis and induce the accumulation of reactive oxygen species (ROS) [15]. Although essential for intercellular communication and defense activation, ROS become destructive once they overwhelm the cellular antioxidant capacity, damaging the photosynthetic apparatus and triggering a self‐reinforcing cycle of oxidative stress and declining performance [16, 17]. These interconnected challenges underscore the need for supplementing the native mechanisms of photosynthetic organisms to improve productivity or stress reporting under specific cultivation conditions.

Biohybrid systems, formed by decorating living systems with non‐biological materials, have emerged as a promising platform to enable new or enhanced functions [18, 19, 20, 21, 22, 23, 24]. For instance, various fluorescent materials such as small‐molecule fluorophores [25, 26], conjugated polymers [27, 28] and carbon dots [29, 30] have been integrated with photosynthetic organisms as artificial antennas to reconfigure sunlight spectra for enhanced photosynthesis. Intracellular hybrid strategies have also been developed, typically by incorporating nanomaterials (e.g., gold nanoparticles [17] and cerium oxide nanoparticles [11, 20]) into photosynthetic organelles or organisms to mitigate ROS and improve stress tolerance. While these approaches enhance biohybrid performance, most operate in a fundamentally monofunctional manner: photosynthesis‐promoting strategies are effective primarily under normal conditions, and stress‐protection strategies usually lack the ability to report environmental changes and dynamically regulate their function. In this context, the integration of light optimization, stress reporting, and stress mitigation is desirable for photosynthetic biohybrids operating in fluctuating environments [20, 31, 32, 33]. The plasma membrane, known as the cell‐environment interface, is ideally positioned to reconfigure incident sunlight while also granting direct access to intracellular ROS signals. Therefore, we hypothesize developing membrane‐anchored fluorescent materials to create a responsive biohybrid interface that couples with the photosynthetic apparatus, reports environmental changes, and enables corresponding functional shifts.

With good biocompatibility and structural flexibility, small‐molecule fluorophores are attractive candidates for the fabrication of biohybrids. Among them, aggregation‐induced emission luminogens (AIEgens) represent a key subclass. Their intramolecular rotors enable restriction‐induced emission upon target binding and facilitate large Stokes shifts via twisted donor–acceptor (D‐A) structures [34, 35, 36]. These features render AIEgens promising artificial components for biohybrid systems, where they can both convert sunlight efficiently and report cellular biophysical states through changes in fluorescence intensity or color [34, 37, 38, 39]. In this work, we thus developed an AIE‐based environmentally responsive antenna TPyB and assembled it onto the plasma membrane of the green microalga *Chlorella pyrenoidosa* (*C. pyre*), constructing a biohybrid with a responsive interface for light optimization and stress management (Figure 1a). TPyB comprises an AIE moiety with a twisted D‐A structure for light‐harvesting and a boronic ester group for sensing H_2_O_2_, a critical ROS that mediates systemic signaling and indicates acute stress [33, 40]. Under normal conditions, the AIE module of TPyB acts as a spectral converter: its large Stokes shift enables the conversion of harmful UV, high‐energy blue, and underutilized green light into photosynthetically favorable red light. Upon stress, rising intracellular H_2_O_2_ oxidatively cleaves the boronic ester group of TPyB, generating another AIEgen TPy. This yields two coordinated benefits: a fluorescence color change from red to yellow that provides a dual‐color reporter for environmental stress, and direct quenching of excess H_2_O_2_ to alleviate oxidative damage. This responsive trait enables the biohybrid to shift from promoting photosynthesis under normal conditions to stress reporting and mitigation, boosting environmental sensing and adaptive response.

**FIGURE 1:**
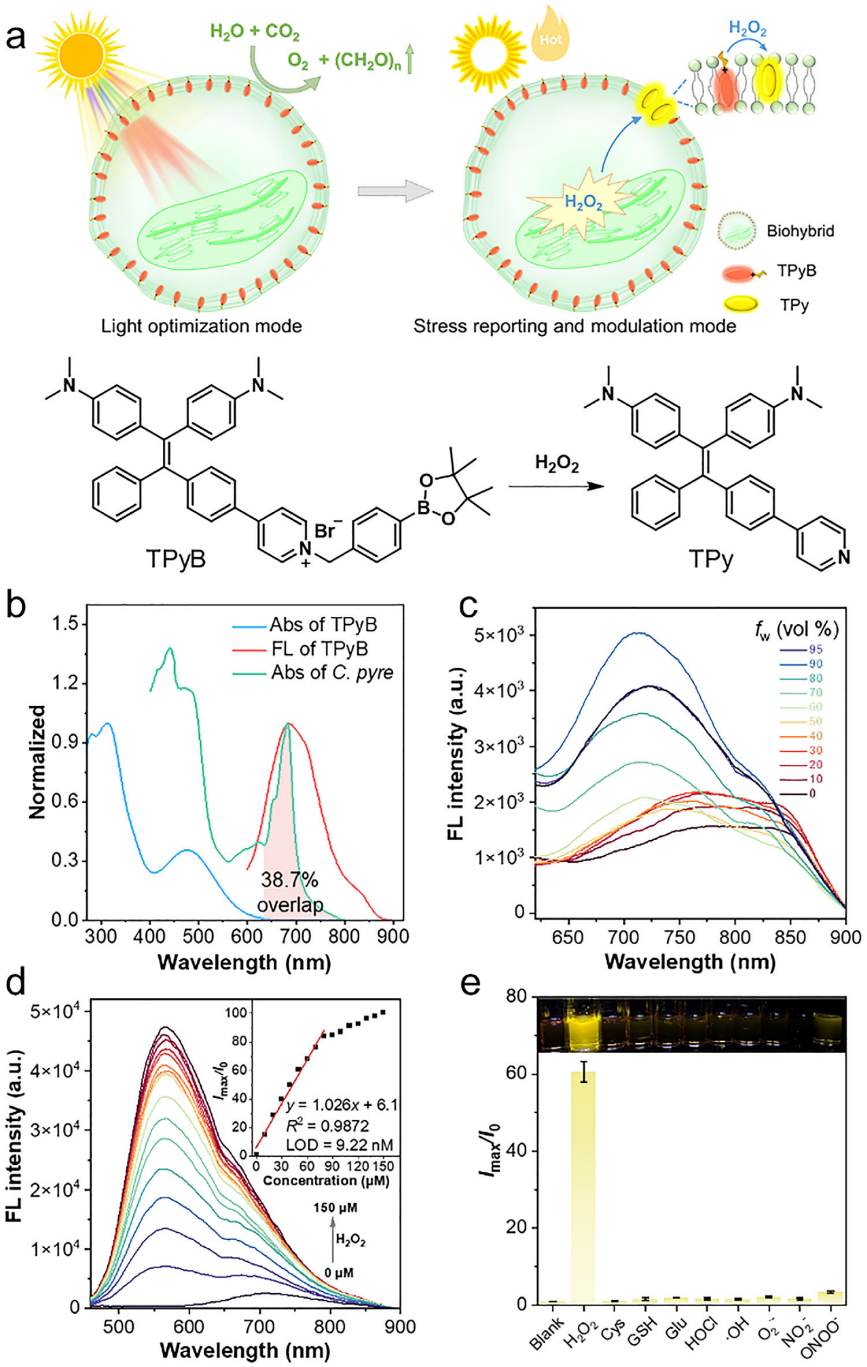
Design and characterization of the environmentally responsive membrane antenna TPyB. (a) Schematic illustration of TPyB assembled onto the algal cell membrane to construct a biohybrid with a responsive interface for light utilization and stress management (top), and H_2_O_2_‐triggered cleavage of TPyB to TPy (bottom). (b) Normalized UV–vis absorption spectrum in DMSO and normalized fluorescence spectrum in BG11 of TPyB (20 µm), *λ*_ex_ = 480 nm, and normalized absorption spectrum of *C. pyre* (OD_680_ = 2.0) in BG11 solution. The pink shading shows the spectral overlap of TPyB emission with algal red absorption. (c) Fluorescence spectra of 20 µm TPyB in DMSO/H_2_O mixtures with different water volume fractions (*f*_w_, vol%), *λ*_ex_ = 480 nm. (d) Fluorescence spectra of 20 µm TPyB upon exposure to H_2_O_2_ (0–150 µm), *λ*_ex_ = 480 nm. Inset: Relative FL intensity (*I*_max_/*I*_0_) versus H_2_O_2_ concentration, where *I*_max_ is the maximum emission intensity of TPyB at a given H_2_O_2_ concentration, and *I*_0_ is the intensity of pure TPyB solution at 566 nm. (e) Relative FL intensity (*I*_max_/*I*_0_) of 20 µm TPyB in the presence of various species (50 µM).

## 2 Results and Discussion

### 2.1 Membrane Antenna Design and Its Photophysical Properties and H_2_O_2_ Responsiveness

It is highly desirable to construct a membrane antenna molecule featuring targeted membrane‐induced emission, a large Stokes shift, and colorimetric H_2_O_2_ responsiveness for light utilization optimization and stress management. To achieve this, we designed TPyB by linking three moieties: a dimethylamino derivative of tetraphenylethylene ((Me_2_N)_2_‐TPE), a pyridinium, and a boronic ester group (Figure 1a). The cationic and amphiphilic nature of TPyB enables targeting of the negatively charged plasma membrane of *C. pyre* via electrostatic and hydrophobic interactions [25]. This membrane binding results in fluorescence turn‐on of the AIE‐active (Me_2_N)_2_‐TPE core, based on the restriction of intramolecular motions mechanism [41]. Furthermore, the (Me_2_N)_2_‐TPE (donor) and pyridinium (acceptor) form a strong D–A pair that promotes a twisted intramolecular charge transfer (TICT) [36], endowing TPyB with a large Stokes shift. Finally, the boronic ester group equips the molecule with specific responsiveness to H_2_O_2_ [42], converting intracellular oxidative signals into optical readouts for stress reporting and response.

TPyB was synthesized according to the route in Figure S1, and its molecular structure was characterized by ^1^H and ^13^C nuclear magnetic resonance (^1^H NMR and ^13^C NMR) and high‐resolution mass spectrometry (HRMS; Figures S2–S4). It possesses broad absorption bands across the UV, blue, and green regions from 300 to 600 nm, and exhibits a red emission band from 600 to 900 nm (Figure 1b). A small HOMO‐LUMO energy gap (Δ*E*) of about 1.577 eV, obtained from the density functional theory (DFT) calculations (Figure S5), leads to a large Stokes shift of about 220 nm. It is worth noting that the emission band of TPyB shows strong overlap with the red light absorption of *C. pyre* (650–750 nm), with an overlap area of 38.7% (Figure 1b), allowing TPyB to act as an artificial antenna that converts high‐energy (UV and blue light) or underutilized photons (green light) into photosynthetically favorable red light. TPyB also shows typical AIE character, as confirmed by the observation that a pronounced increase in fluorescence intensity was observed for TPyB in DMSO/water mixtures with 90% water fraction due to the occurrence of aggregation (Figure 1c and Figure S6). A slight blue shift was also observed upon aggregation, consistent with its D‐A structure (Figure 1c). The AIE character of TPyB would favor the membrane binding‐induced emission for light conversion.

The H_2_O_2_ responsiveness of TPyB was then investigated. After exposure to H_2_O_2_, the 4‐methylphenylboronic acid pinacol ester in TPyB was cleaved to generate TPy (Figure 1a), as confirmed by mass spectrometry (Figure S7). TPy is also AIE‐active, as evidenced by a marked fluorescence increase in DMSO/water mixtures with a 70% water fraction (Figure S8). DFT calculations showed that compared to TPyB, the spatial overlap between the donor‐localized HOMO and acceptor‐localized LUMO of TPy increased (Figure S5), indicating a decreased charge transfer effect. Therefore, upon H_2_O_2_‐triggered oxidative cleavage, the D‐A interaction of TPyB is attenuated, inducing a distinct hypsochromic shift in fluorescence from red (∼710 nm) to yellow (∼566 nm) along with enhanced intensity (Figure 1d). This spectral response and AIE behavior of TPyB before and after H_2_O_2_ responsiveness make it a dual‐color reporter for H_2_O_2_, which facilitates the sensitive monitoring of H_2_O_2_ [42]. The limit of detection (LOD) was calculated as 9.2 nM, based on the increase in relative fluorescence intensity (*I*_max_/*I*_0_) of TPyB with rising H_2_O_2_ concentration (Figure 1d). Further, the result in Figure 1e showed that only H_2_O_2_ enhanced the fluorescence intensity at 566 nm, whereas other ROS or reactive nitrogen species (RNS) and common biomolecules (such as cysteine (Cys), glucose (Glu), and glutathione (GSH)) caused negligible responses, indicating the high selectivity for H_2_O_2_. These results establish TPyB as a sensitive reporter for intracellular H_2_O_2_ levels. Collectively, TPyB combines AIE‐based light harvesting with H_2_O_2_ responsiveness, acting as a promising biohybrid component for microalgae that could optimize photosynthesis under normal conditions while providing stress reporting and modulation.

### 2.2 Membrane Localization on Microalgae and Biocompatibility

To construct the photosynthetic biohybrid, we chose the green microalga *C. pyre* as a model organism. Confocal laser scanning microscopy (CLSM) images revealed that TPyB‐treated *C. pyre* cells exhibited red fluorescence at the cell periphery, suggesting the association of TPyB with the algal cell membrane (Figure S9). To confirm this localization, we co‐stained *C. pyre* with TPyB and the membrane‐specific probe, 1,1'‐dioctadecyl‐3,3,3',3'‐tetramethylindocarbocyanine perchlorate (DiI; Figure 2a and Figure S10). The merged CLSM image and intensity profiles showed strong overlap between fluorescence signals of TPyB and DiI (Figure 2a). Further, to exclude the possibility of TPyB retention in the cell wall, we compared the TPyB fluorescence intensity in *C. pyre* with or without cell wall removal after TPyB pre‐treatment (Figure S11). The comparable intensities between the two groups indicate that TPyB is not retained in the cell wall. These results confirm that TPyB is specifically localized on the algal plasma membrane. Flow cytometry analysis (Figure 2b) supported the prevalence of this binding, with 93.2% of TPyB‐treated cells shifting to the double‐positive quadrant (Q2), compared to untreated controls that were in the single‐positive region (Q3). To quantitatively characterize the interaction, we performed isothermal titration microcalorimetry (ITC). Titration of TPyB into algal suspensions produced a sigmoidal binding isotherm (Figure 2c and Figure S12). The strongly exothermic enthalpy change (Δ*H*_obs_) gradually decreased upon successive additions, indicating progressive saturation of accessible binding sites on the cell. The binding constant (*K* = 2.15 × 10^5^ M^−1^) reflects a high affinity of TPyB for *C. pyre* cells. The exothermic Δ*H*_obs_ (Δ*H* < 0) and the concomitant entropy decrease (Δ*S* < 0) indicate a binding process of TPyB and algal cells driven by electrostatic attraction, followed by ordered insertion into the hydrophobic membrane [43]. This is further supported by zeta potential results (Figure 2d), which showed no significant shift upon TPyB binding, confirming that TPyB inserts into the algal cell membrane without shielding the surface charges [25, 44].

**FIGURE 2:**
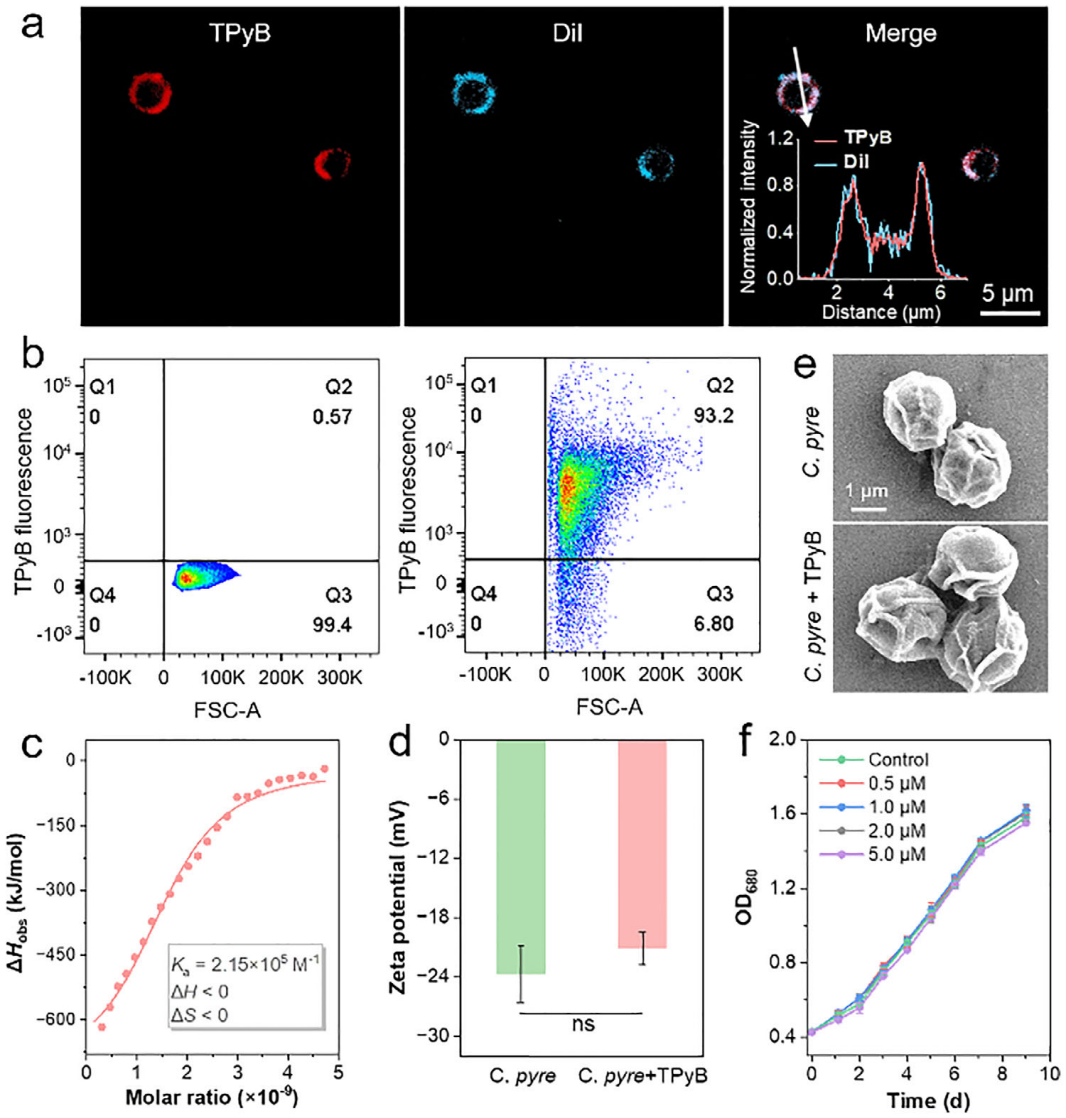
Construction of a biohybrid with an artificial membrane component. (a) CLSM images of *C. pyre* co‐stained with 20 µm TPyB and 10 µm DiI. *λ*_ex_ = 543 nm and *λ*_em_ = 580–650 nm for TPyB, *λ*_ex_ = 543 nm and *λ*_em_ = 550–580 nm for DiI (false‐colored blue). Inset: Intensity profiles of TPyB and DiI along a line crossing a representative cell. (b) Fluorescence‐activated cell sorting analysis of *C. pyre* without (left) and with (right) 20 µm TPyB treatment. (c) Variation in observed enthalpy change (Δ*H*_obs_) upon titrating 150 µm TPyB into *C. pyre* suspensions (OD_680_ = 1.0). (d) Zeta potential of *C. pyre* before and after treatment with 20 µm TPyB. Error bars: mean ± SD (*n* = 3). *P* > 0.05, ns, not significant. (e) SEM images of *C. pyre* cells without and with 20 µm TPyB. (f) Growth curves of *C. pyre* without and with different concentrations of TPyB. Error bars: mean ± SD (*n* = 3).

Following its membrane localization, we evaluated the potential biotoxicity of TPyB toward *C. pyre* cells. Scanning electron microscopy (SEM) images showed that algal cell morphology remained intact after TPyB treatment, with no visible surface damage (Figure 2e). The growth of *C. pyre* under different TPyB concentrations further confirmed that TPyB did not inhibit algal proliferation (Figure 2f). These findings demonstrate good biocompatibility of TPyB, supporting its suitability as an artificial membrane‐integrated component in the biohybrid system.

### 2.3 Membrane Antenna for Light Optimization and Photosynthesis Augmentation

As a membrane antenna, the light‐harvesting and energy‐transfer ability of TPyB was first explored spectroscopically. Under blue light excitation, the red emission of TPyB was quenched upon addition of *C. pyre*, accompanied by an increase in chlorophyll‐associated fluorescence (Figure S13a). Given that the distance between the algal membrane and chlorophyll far exceeds the Förster resonance energy transfer range [45], this spectral change suggests a radiative energy transfer process: TPyB absorbs blue light and emits red light, which is then reabsorbed by algal chlorophylls due to the large spectral overlap of the red emission of TPyB with the red absorption of *C. pyre* (Figure 1b). Similar results were observed under UV and green light excitation (Figure S13b,c). These results indicate that TPyB can serve as a membrane antenna, capturing UV, blue, and green photons and channeling them to the algal photosynthetic apparatus via red light emission. To determine whether this spectral conversion enhances photochemistry, we quantified photosynthetic electron generation by monitoring the absorbance decrease at 600 nm of the artificial electron acceptor 2,6‐dichlorophenolindophenol (DCPIP) [46]. In the absence of TPyB, the electron generation rates of *C. pyre* under UV, blue or green light were substantially lower than under white light (Figure 3a and Figure S14). Upon addition of TPyB, the electron generation rates under all three wavelengths increased markedly, with UV and blue light rates reaching or even surpassing the level of algae alone under white light. This provides direct evidence that the TPyB‐mediated light conversion effectively drives photosynthetic electron transport. Consequently, under white light irradiation, TPyB boosted the electron generation rate of algae by 66.7% (Figure 3a and Figure S14a).

**FIGURE 3:**
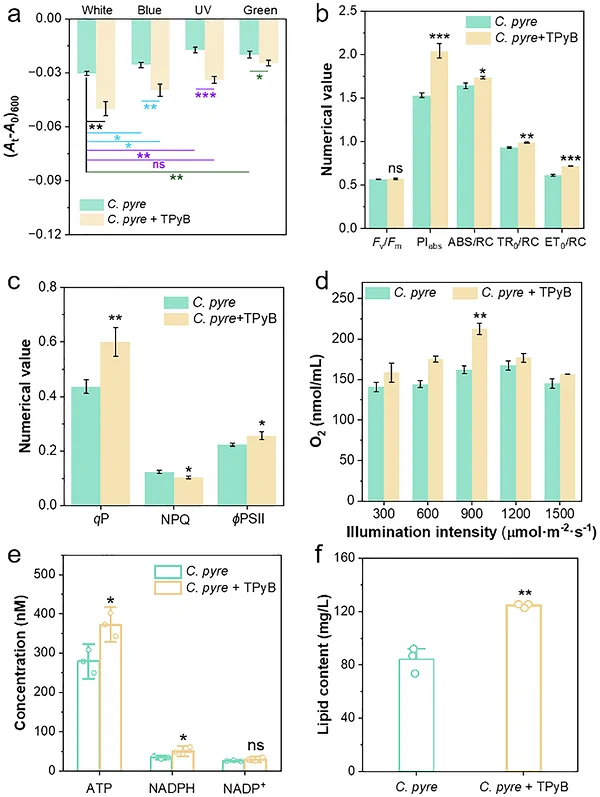
Optimized light utilization and enhanced photosynthesis. (a) Absorbance change at 600 nm of DCPIP in *C. pyre* suspension without and with 0.5 µm TPyB after 5 min of illumination under white (900 µmol·m^−2^·s^−1^), blue (400 µmol·m^−2^·s^−1^), UV (4.6 µmol·m^−2^·s^−1^), or green light (400 µmol·m^−2^·s^−1^). (b) Dark‐adapted and (c) light‐adapted chlorophyll fluorescence parameters of *C. pyre* in the absence and presence of 0.5 µm TPyB. (d) Oxygen evolution in *C. pyre* without and with 0.5 µm TPyB under varying white light intensities over 1500 s. (e) ATP, NADPH and NADP^+^ contents in *C. pyre* without and with 0.5 µm TPyB after 30 min white light irradiation (900 µmol·m^−2^·s^−1^). (f) Lipid content in *C. pyre* without and with 0.5 µm TPyB after 12 days of cultivation. Data are presented as mean ± SD (*n* = 3). Statistical significance: *P* > 0.05, ns, not significant; ^*^ *p* < 0.05, significant; ^**^ *p* < 0.01 and ^***^ *p* < 0.01, highly significant.

We further investigated the effect of this optimized light input on photosystem II (PSII) function. The chlorophyll fluorescence kinetics curves and the obtained chlorophyll fluorescence analysis under dark adaptation revealed significant increases in energy absorption per reaction center (ABS/RC), trapping (TR_o_/RC), and electron transport (ET_o_/RC) parameters (Figure 3b and Figure S15), reflected by a 33.3% rise (from 1.531 to 2.041) in the performance index for absorbed light (PI_ABS_). Under light adaptation, the photochemical quenching coefficient (*qP*) of *C. pyre* cells increased by 37.3%, while NPQ decreased by 16.2% (Figure 3c), indicating a greater fraction of absorbed light is directed toward photochemistry rather than dissipated as heat. Although the maximum quantum yield of PSII (*F*_v_/*F*_m_) did not obviously increase (Figure 3b), the actual quantum yield (*ϕ*PSII) increased by 15.2% (from 0.223 to 0.257, Figure 3c). In addition, control experiments with TPyB alone showed negligible fluorescence signal compared with that of *C. pyre* cells with or without TPyB treatment (Figure S15), ruling out the potential interference from TPyB fluorescence. These results collectively confirm that TPyB reshapes the sunlight into more photosynthetically efficient wavelengths, improving the operational efficiency of PSII across light absorption, capture, and utilization.

The enhanced photochemical performance of PSII in the biohybrid translated into elevated outputs of the light and dark reactions. TPyB itself did not produce oxygen (Figure S16a). Compared with algae alone, the biohybrid showed a 31.2% increase in oxygen evolution from 162.1 to 212.7 nmol/mL under optimal conditions (0.5 µm TPyB, 900 µmol·m^−2^·s^−1^; Figure 3d and Figure S16b). Consistent with this, cellular ATP (adenosine triphosphate) and NADPH (reduced nicotinamide adenine dinucleotide phosphate) levels rose by 33.5% and 47.2%, respectively (Figure 3e), indicating greater availability of energy and reducing power to drive the dark reactions. To confirm this, we measured the activity of Rubisco, a key enzyme for carbon fixation and biomass synthesis [47]. Rubisco activity increased by 43.2% from 2.20 to 3.15 nmol·min^−1^(10^8^ cells)^−1^ in TPyB‐treated cells (Figure S17), reflecting an accelerated capacity for carbon fixation and biomass synthesis. This led to a 47.9% increase in lipid content (from 84.1 to 124.4 mg/L) after 12 days of cultivation (Figure 3f). To clarify whether the observed photosynthesis enhancement also originates from free TPyB in solution, we performed the following control experiments. As an AIE‐active molecule, TPyB exhibits strong fluorescence only when its intramolecular motion is physically restricted, whereas free TPyB in solution remains as dispersed monomers with weak emission, rendering its light‐conversion efficiency negligible. To verify this, we removed unbound TPyB by centrifugation after TPyB treatment; the washed biohybrid exhibited O_2_ evolution comparable to the unwashed control (Figure S18), confirming that free TPyB contributes negligibly to the observed enhancement. These results support that the photosynthesis enhancement is primarily attributable to the membrane‐associated TPyB. Together, TPyB functions as a membrane antenna to create an assembled biohybrid interface that enhances light utilization and PSII performance, significantly boosting the photosynthesis of the biohybrid under normal conditions.

### 2.4 Stress Reporting and Tolerance Enhancement

The membrane‐localized TPyB, with its H_2_O_2_ responsiveness and good photothermal stability (Figure S19), is expected to enable the biohybrid to monitor environmental stress by converting intracellular oxidative signals into optical readouts. To confirm this, we first visualized the in situ H_2_O_2_ responsiveness of TPyB in the biohybrid by CLSM imaging. As shown in Figure 4a, TPyB‐treated *C. pyre* cells displayed red fluorescence on the algal membrane. In addition, no fluorescence signal was detected in the channel corresponding to the cleavage product TPy, indicating that the possible spectral shifts of TPyB upon membrane binding due to its D‐A structure do not interfere with the H_2_O_2_‐responsive signal. Upon addition of H_2_O_2_, the red emission decreased concurrently with the appearance of a yellow signal, confirming the conversion of TPyB to its cleaved product TPy. It was observed that despite lacking cationic charge, TPy still remained membrane‐associated, possibly attributed to its in situ generation at the algal membrane and residual hydrophobic interactions with the lipid domain, as confirmed by the absence of membrane localization when neutral TPy was applied alone to algae cells (Figure S20). The reaction kinetics were further monitored by tracking TPy emission at 570 nm. In the biohybrid, the addition of H_2_O_2_ triggered a time‐dependent emission increase, which nearly plateaued at 2 h (Figure S21a,b). In contrast, algae alone showed no such response (Figure S21a,c). Flow cytometry further provided population‐level validation. After H_2_O_2_ exposure, the fluorescence of TPy‐positive cells increased progressively over time (Figure S22a). After 2 h, the proportion of cells with TPy fluorescence above the mean intensity increased from 17.4% to 50.8% (Figure S22b). Moreover, 62.3% of the cell population exhibited a distribution shift (Figure 4b), indicating widespread activation of the reaction of TPyB with H_2_O_2_ across the cell population. In addition, we also evaluated the biosafety of the reaction product TPy by testing its effect on algal growth. No inhibition was observed across the tested concentrations (Figure S23), confirming that the H_2_O_2_ sensing of TPyB does not produce cytotoxic by‐products. Thus, TPyB, as a biocompatible and H_2_O_2_‐responsive membrane component, potentially endows the biohybrid with a responsive interface for monitoring environmental stress.

**FIGURE 4:**
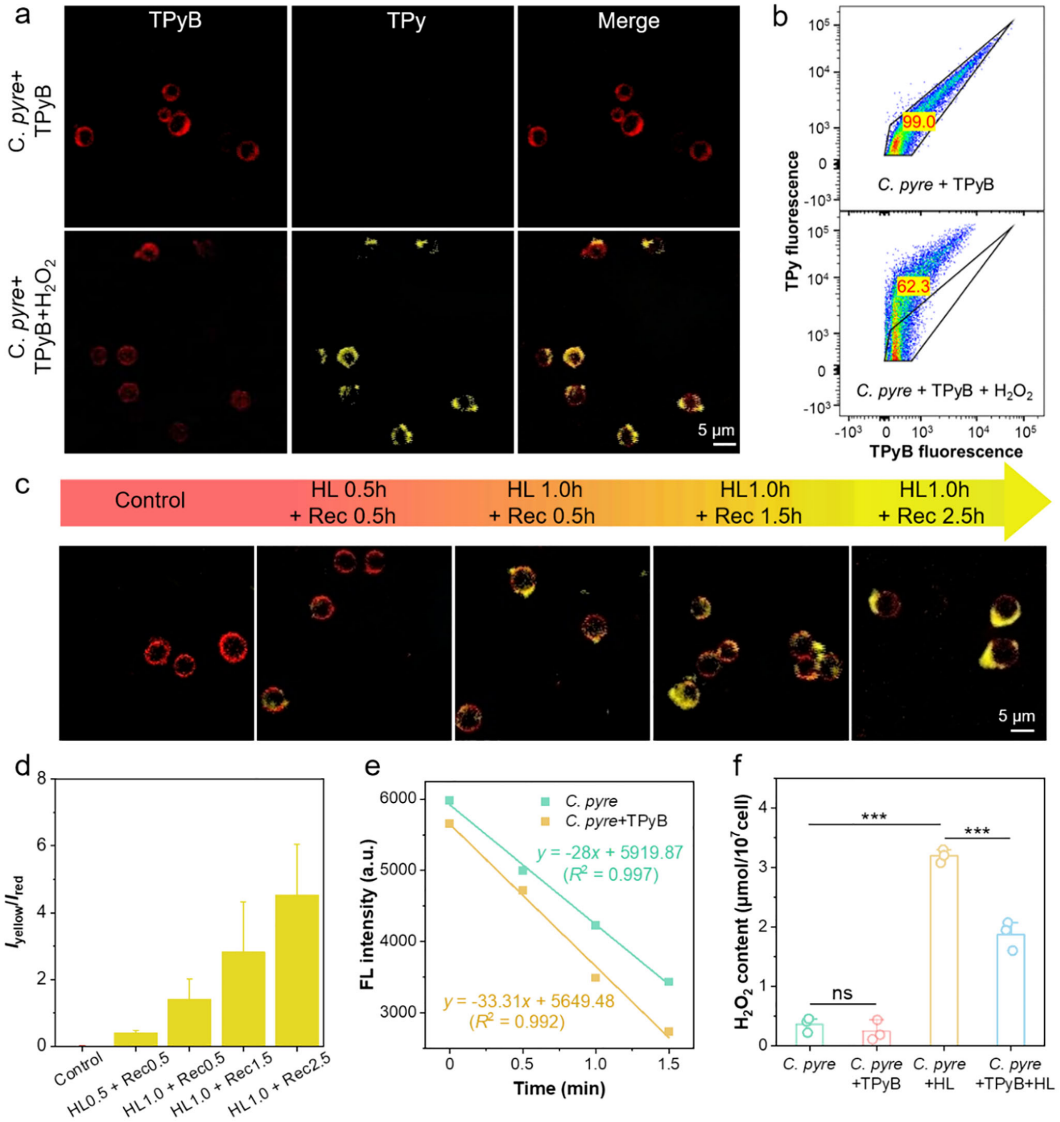
In situ H_2_O_2_ response of TPyB for environmental stress reporting. (a) CLSM images under different treatments: (top) 20 µm TPyB alone and (bottom) 20 µm TPyB with 100 µm H_2_O_2_ after 90 min incubation. Imaging conditions: *λ*_ex_ = 543 nm and *λ*_em_ = 600–650 nm for TPyB; *λ*_ex_ = 405 nm and *λ*_em_ = 450–550 nm for TPy. (b) Flow cytometric analysis of the *C. pyre*/TPyB biohybrid after 120 min reaction with 100 µm H_2_O_2_. (c) CLSM images and (d) fluorescence intensity ratio (*I*_yellow_/*I*_red_) in *C. pyre* cells after different times of HL exposure (2000 µmol·m^−2^·s^−1^) and then recovery (Rec) for varying times in the presence of TPyB (20 µm). The biohybrid without HL exposure served as the control. Imaging conditions: *λ*_ex_ = 543 nm and *λ*_em_ = 600–650 nm for TPyB; *λ*_ex_ = 405 nm, *λ*_em_ = 450–550 nm for TPy. (e) Fluorescence intensity changes at 530 nm of DCFH‐DA (50 µm) over recovery time in *C. pyre* and biohybrid after 1 h of HL exposure. [TPyB] = 1.0 µm. (f) Intracellular H_2_O_2_ levels in *C. pyre* and biohybrid with or without 1 h of HL exposure, followed by 2 h of recovery. Error bars: mean ± SD (*n* = 3). *P* > 0.05, ns, not significant; ^***^ *p* < 0.001, highly significant.

To validate this, we subjected *C. pyre* cells to HL (2000 µmol·m^−2^·s^−1^), a representative source of photosynthetic oxidative damage [12]. As shown in Figure 4c, after HL exposure, the red fluorescence of membrane‐integrated TPyB decreased while yellow emission increased, indicating intracellular H_2_O_2_‐mediated cleavage of TPyB to TPy. Quantitative analysis of the fluorescence ratio (*I*_yellow_/*I*_red_) on the cell membrane showed a progressive increase with longer HL exposure and extended recovery time (Figure 4d). This spectrally resolvable shift confirms that TPyB enables the reporting of HL stress by sensing intracellular H_2_O_2_.

Importantly, the increase in the *I*_yellow_/*I*_red_ of TPyB also indicates that the stress‐reporting process is coupled with H_2_O_2_ scavenging. We thus monitored intracellular ROS levels using the 2’,7’‐dichlorofluorescein diacetate (DCFH‐DA) probe in algae with and without TPyB [48]. In algae alone, HL exposure for 1 h caused intense probe fluorescence, indicating substantial ROS accumulation; the fluorescence gradually declined with prolonged recovery time, reflecting the reduction of ROS by endogenous defenses (Figure 4e and Figure S24a). In the biohybrid, the probe fluorescence was lower after 1 h of HL exposure and decreased more rapidly over recovery time (Figure 4e and Figure S24b), verifying that TPyB effectively mitigates intracellular H_2_O_2_ accumulation. Consistent with this, direct measurement of intracellular H_2_O_2_ content confirmed that the biohybrid maintained markedly lower H_2_O_2_ levels than algae alone after 1 h of HL stress and 2 h of recovery (Figure 4f). These results collectively demonstrate that the membrane‐integrated TPyB can act as a stress reporter via a spectrally resolvable fluorescence shift while concurrently reducing the oxidative load through H_2_O_2_ consumption. This integrated sense‐and‐respond capability allows the biohybrid to monitor and alleviate stress, facilitating cellular tolerance under adverse conditions.

To test that the H_2_O_2_ scavenging ability of TPyB directly confers photosynthetic protection, we first exposed algal cells to exogenous H_2_O_2_ and then assessed their photosynthetic performance. As shown in Figure S25, the biohybrid maintained significantly higher *F*_v_/*F*_m_ than algae alone after H_2_O_2_ treatment, even reaching levels comparable to unstressed controls, indicating preserved PSII integrity [49]. This was further supported by the fact that less impaired oxygen evolution was observed in the biohybrid (Figure S26). Since the H_2_O_2_‐induced cleavage product TPy itself does not enhance photosynthesis (Figure S27), this protective function on the photosynthetic apparatus can be attributed to the H_2_O_2_‐consuming ability of TPyB. We next examined whether this protective function translates into enhanced tolerance under stress conditions such as HL (2000 µmol·m^−2^·s^−1^) and HT (40°C). Following 1 h of HL or HT exposure, the biohybrid exhibited less impairment and better recovery of both *F*_v_/*F*_m_ and *ϕ*PSII than algae alone (Figure 5a,b). Accordingly, the biohybrid sustained higher oxygen evolution after HL stress, maintaining levels comparable to unstressed controls, whereas algae alone showed persistently depressed oxygen output even after 2 h of recovery (Figure 5c). Similar oxygen results were observed under HT stress (Figure S28). The chlorophyll content measurements further supported this (Figure S29). After HL stress, the algae alone showed a significant decrease (about 8.2%), while the biohybrid maintained chlorophyll levels comparable to unstressed controls, confirming the photoprotective role of TPyB in mitigating photooxidative damage. These results indicate that by scavenging H_2_O_2_, TPyB effectively enhances the capacity of algal cells to withstand and recover from adverse environmental conditions.

**FIGURE 5:**
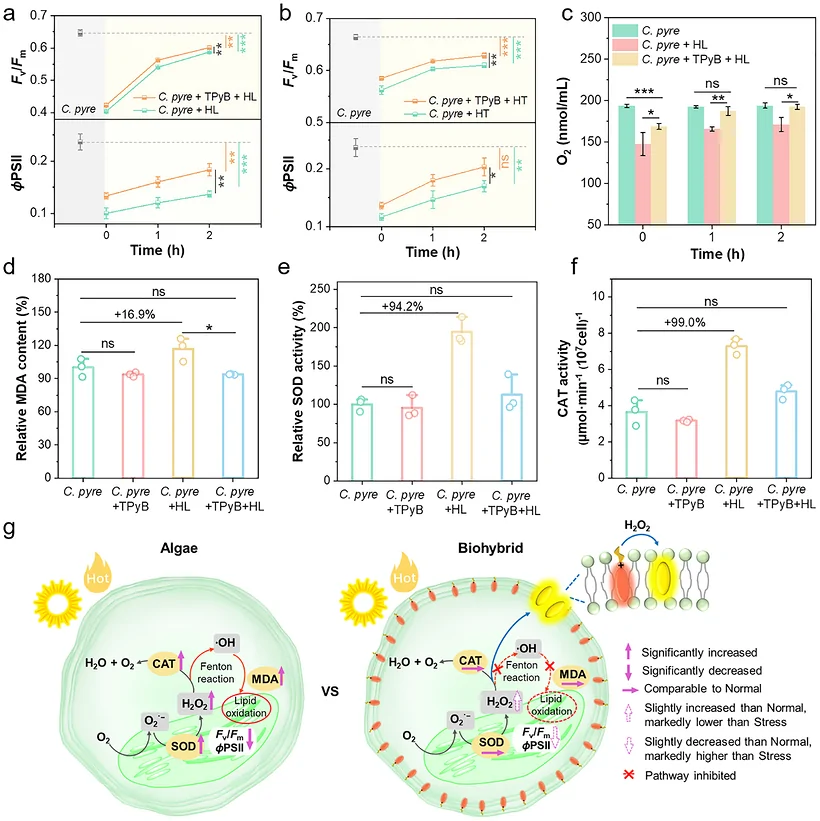
H_2_O_2_ scavenging capability of TPyB for enhanced tolerance in biohybrid. (a) *F*_v_/*F*_m_ and *ϕ*PSII parameters of *C. pyre* and the *C. pyre*/TPyB biohybrid after 1 h of HL exposure (2000 µmol·m^−2^·s^−1^) and subsequent recovery for different times. (b) *F*_v_/*F*_m_ and *ϕ*PSII of *C. pyre* and the biohybrid after 1 h of HT exposure (40°C) and subsequent recovery for different times. (c) Oxygen evolution under white light (900 µmol·m^−2^·s^−1^) over 1500 s in *C. pyre* and the biohybrid after 1 h of HL exposure and subsequent recovery for different times. (d) Relative MDA content, (e) relative SOD activity, and (f) CAT activity in *C. pyre* and the biohybrid, without and with 1 h of HL exposure followed by 2 h of recovery. (g) Schematic illustration of TPyB‐mediated stress alleviation and enhanced tolerance under adverse conditions. Data are presented as mean ± SD (*n* = 3). *P* > 0.05, ns, not significant; ^*^ *p* < 0.05, significant; ^**^ *p* < 0.01 and ^***^ *p* < 0.001, highly significant.

It has been reported that under stress, electron leakage in the photosynthetic electron transport chain of chloroplasts generates superoxide anion (O_2_^−^) by reducing O_2_. O_2_^−^ is rapidly converted into H_2_O_2_ by superoxide dismutase (SOD) within the chloroplast. H_2_O_2_ diffuses into the cytoplasm, where some is scavenged by catalase (CAT) or peroxidases, and the remainder generates highly reactive hydroxyl radicals (·OH) via the Fenton reaction [15]. These radicals attack thylakoid and other cellular membranes, triggering lipid peroxidation chain reactions that lead to malondialdehyde (MDA) accumulation and membrane dysfunction [50], as illustrated in Figure 5g. Therefore, to further clarify the enhanced tolerance of the biohybrid, we evaluated the biochemical antioxidant response. Following HL or HT stress, algae alone exhibited increased MDA levels (16.9% under HL, 19.3% under HT; Figure 5d and Figure S30), indicating substantial oxidative damage. In parallel, a significant up‐regulation of SOD and CAT activities was observed (Figure 5e,f and Figure S31). Despite this robust enzymatic response (e.g., SOD and CAT activities increased by ∼94% and ∼99% under HL), the photosynthetic performance of algae was significantly compromised (Figure 5a–c). This suggests that the endogenous defenses are insufficient to cope with these abiotic stresses. By contrast, the TPyB‐integrated biohybrid maintained SOD and CAT activities at levels statistically comparable to unstressed controls (Figure 5e,f and Figure S31), while keeping MDA content at baseline (Figure 5d and Figure S30). This indicates that the membrane‐integrated TPyB prevents a major oxidative perturbation, thereby pre‐empting the need for large‐scale induction of antioxidant enzymes. Anchored on the plasma membrane, TPyB efficiently scavenges H_2_O_2_ that diffuses to its vicinity (Figure 5g). This scavenging action not only removes the substrate that triggers the Fenton reaction but also generates a thermodynamic driving force: as H_2_O_2_ is rapidly consumed, the upstream SOD‐catalyzed reaction proceeds continuously to the right, promoting the conversion of O_2_^·−^ into H_2_O_2_. As a result, the algal cell is protected from oxidative damage without the substantial metabolic cost of mounting an antioxidant defense system. Thus, by quenching stress‐induced intracellular H_2_O_2_, TPyB allows the algal cell to actively maintain redox homeostasis and achieve greater stress tolerance. This integrated sense‐and‐respond functionality shifts the algal stress‐coping strategy from passive damage containment toward active adaptation and accelerated recovery.

## 3 Conclusion

In summary, we have developed an environmentally responsive antenna TPyB, and assembled it onto the algal cell membrane to construct a biohybrid with a responsive interface for light optimization and stress management. Under normal conditions, TPyB functions as a spectral converter, transforming harmful UV and underutilized visible light into photosynthetically favorable red emission, which boosts photosynthesis and biomass (e.g., 47.9% increase in lipid yield). Upon stress‐induced H_2_O_2_ accumulation, TPyB undergoes oxidative cleavage, triggering a fluorescence color change (red to yellow) for stress reporting and direct ROS quenching. As a result, the biohybrid maintains redox homeostasis without mounting a costly antioxidant defense, achieving faster recovery and enhanced tolerance. Therefore, by assembling a responsive membrane antenna with living organisms, the biohybrid can conditionally shift from light optimization to stress management mode. Furthermore, from a practical application perspective, some key factors should be considered for sustaining biohybrid performance across multiple growth cycles. First, although the AIE character of TPyB confers good photostability, prolonged illumination may still cause gradual photobleaching or membrane dissociation. Second, repeated cell divisions would progressively dilute the membrane‐associated TPyB, which is also consumed during each stress response. Future work could therefore evaluate the antenna molecule retention over repeated subcultures and explore periodic replenishment as a strategy to restore its light‐optimization and stress‐reporting functions. These considerations will be important for translating this biohybrid from a proof‐of‐concept towards practical applications. Overall, this work establishes a bioaugmentation platform that reforms the design paradigm of biohybrids from static, single‐function enhancement to dynamic, responsive integration, paving the way for advanced biohybrids capable of proactively sensing, reporting, and adapting to environmental fluctuations.

## Author Contributions

**Bing Liu**: methodology, investigation, validation, data curation, Writing – original draft. **Yuchun Han**: methodology, resources. **Gongmeiyue Su**: methodology, investigation, data curation, validation, writing – original draft. **Zhao Li**: supervision, funding acquisition, writing – review and editing, methodology, validation, resources, formal analysis. **Chengcheng Zhou**: conceptualization, methodology, data curation, validation, formal analysis, supervision, funding acquisition, project administration, writing – original draft, writing – review and editing, resources. **Hang Yao**: methodology, resources. **Jingyi Shi**: investigation, data curation.

## Conflicts of Interest

The authors declare no conflicts of interest.

## Supporting information




**Supporting File**: advs77205‐sup‐0001‐SuppMat.docx.

## Data Availability

The data that support the findings of this study are available in the supplementary material of this article.
