# Supplementary material for: Responsive Membrane Antenna Enables Adaptive Biohybrid Interface for Photosynthesis Promotion, Stress Reporting and Modulation
Source: Adv Sci (Weinh). 2026 Aug 15:e77205. Online ahead of print. doi: 10.1002/advs.77205 (PMC13477250; doi:10.1002/advs.77205)
Supplement: Supplementary file 1 [file ADVS-9999-e77205-s001.docx]

Supporting Information

**Responsive Membrane Antenna Enables Adaptive Biohybrid Interface for Photosynthesis Promotion, Stress Reporting and Modulation**

Bing Liu^#[a]^, Gongmeiyue Su^#[b]^, Jingyi Shi,^[a]^ Hang Yao^[a]^, Yuchun Han^[c]^, Zhao Li*^[b]^ and Chengcheng Zhou*^[a]^

^[a]^ Jiangsu Provincial Key Laboratory of Green & Functional Materials and Environmental Chemistry, School of Chemistry and Materials, Yangzhou University, Yangzhou, 225002, China

^[b]^ School of Medical Science and Engineering, School of Materials Science and Engineering, Beijing Institute of Technology, Beijing 100081, China

^[c]^ CAS Key Laboratory of Colloid, Interface and Chemical Thermodynamics, Institute of Chemistry, Chinese Academy of Sciences, Beijing 100190, P. R. China. University of Chinese Academy of Sciences,
Beijing 100049, P. R. China

^#^These authors contributed equally to this work

*Correspondence email: lizhao@bit.edu.cn, zhoucc@yzu.edu.cn

**Materials and Instruments**

The raw chemicals and organic solvents for molecular synthesis were purchased from Shanghai Titan Scientific Co. Ltd. and used as received. 1,1'-Dioctadecyl-3,3,3',3'-tetramethylindocarbocyanine perchlorate (DiI), plant protoplasts isolation kit, enhanced ATP assay kit, enhanced NADP^+^/NADPH assay kit, catalase (CAT) assay kit, total superoxide dismutase (SOD) assay kit with AST-8 and lipid peroxidation MDA assay kit were purchased from Beyotime. Rubisco activity assay kit and Hydrogen peroxide (H_2_O_2_) content assay kit were purchased from Solarbio. 2’,7’-Dichlorofluorescein diacetate (DCFH-DA, 97%) was obtained from Energy Chemical. 2,6-Dichlorophenolindophenol (DCPIP, 97%) was purchased from Macklin. Hydrogen peroxide (30% aqueous solution) was obtained from Sinopharm Chemical Reagent Co., Ltd. Phosphate buffered saline (1× PBS, pH 7.4) was obtained from WISENT. *Chlorella pyrenoidosa* (*C. pyre*, FACHB-9) and BG11 medium were obtained from Freshwater Algae Culture Collection at the Institute of Hydrobiology, FACHB. *C. pyre* cells were incubated in an illumination incubator (BIC-250, Shanghai Boxun Industrial Co. Ltd., China). ^1^H NMR and ^13^C NMR spectra were measured on a Bruker spectrometer (AVANCE III, Bruker, Germany). MS measurements were performed in a positive ion mode on a Mass Spectrometer (QTRAP5500, AB Sciex, USA). UV-vis absorption and fluorescence spectra were measured on a spectrophotometer (UV-2700i, Shimadzu, Japan) and a spectrofluorometer (F7000, Hitachi, Japan), respectively. Size distribution and zeta potential results were measured on Nano ZS (ZEN3690, Malvern). Fluorescence images were collected on a confocal laser scanning microscopy (LSM 880NLO, Carl Zeiss, Germany). Isothermal titration microcalorimetry (ITC) measurements were conducted on a TAM III (TA) instrument. Flow cytometric analysis was performed on a flow cytometry (LSRFortessa^TM^, BD, USA). Oxygen evolution was measured on liquid-phase oxygen electrode systems (Chlorolab-2, Hansatech, UK). Chlorophyll parameters were tested using Chlorophyll fluorescence monitor (Aquapen AP 110-C, Photon Systems Instruments, Czech Republic). ATP, NADPH and MDA content, SOD and CAT activity were measured on a microplate reader (Spark, Tecan, Switzerland).

**Experimental methods**

**Synthesis of TPy.** 4,4’-(2-(4-Bromophenyl)-2-phenylethene-1,1-diyl)bis(*N*,*N*-dimethylaniline) (497.47 mg, 0.10 mmol) and pyridin-4-ylboronic acid (122.92 mg, 0.10 mmol) were placed in a 50 mL round-bottom flask. 20 mL deoxygenated THF and 4 mL 2 M K_2_CO_3_ were added into the flask under nitrogen. The reaction mixture was heated at reflux under argon for 16 h. After evaporating the solvent, the crude product was purified by silica gel column chromatography using DCM/MeOH (100 : 1, *V*/*V*) as eluent. The final product was dried to get a yellow solid with a 69% yield. ^1^H NMR (600 MHz, DMSO-*d*_6_), *δ* (ppm): 8.58 (d, *J* = 5.3 Hz, 2H), 7.64 (dd, *J* = 29.7, 6.4 Hz, 4H), 7.14 (t, *J* = 7.5 Hz, 2H), 7.07 (d, *J* = 7.8 Hz, 3H), 6.98 (d, *J* = 7.6 Hz, 2H), 6.78 (dd, *J* = 28.2, 8.4 Hz, 4H), 6.46 (dd, *J* = 18.8, 8.4 Hz, 4H), 2.83 (s, 12H). ^13^C NMR (151 MHz, DMSO-*d*_6_), *δ* (ppm): 150.21, 148.80, 148.69, 144.64, 141.92, 135.18, 131.97, 131.94, 131.74, 131.00, 130.95, 127.88, 125.99, 125.87, 120.68, 111.28, 111.14, 40.06. HR-MS (m/z): calculated for C_35_H_33_N_3_ [M+H]^+^: 496.2747, found: 496.2742. These characterization results are shown in Figure S32-S34.

**Synthesis of TPyB.** TPy (49.53 mg, 0.01 mmol) and 2-(4-(bromomethyl)phenyl)-4,4,5,5-tetramethyl-1,3,2-dioxaborolane (29.7 mg, 0.01 mmol) were placed in a 25 mL round-bottom flask. 10mL ACN was added into the flask. The reaction mixture was heated at reflux overnight. After evaporating the solvent, the crude product was purified by silica gel column chromatography using DCM/MeOH (10 : 1, *V*/*V*) as eluent. The final product was dried to get a dark red solid with a 56% yield. ^1^H NMR (600 MHz, DMSO-*d*_6_), *δ* (ppm): 9.11 (d, *J* = 6.5 Hz, 2H), 8.46 (d, *J* = 7.2 Hz, 2H), 7.88 (d, *J* = 8.6 Hz, 2H), 7.73 (d, *J* = 8.0 Hz, 2H), 7.51 (d, *J* = 7.8 Hz, 2H), 7.17 – 7.09 (m, 5H), 6.98 (d, *J* = 6.8 Hz, 2H), 6.81 (d, *J* = 8.8 Hz, 2H), 6.77 (d, *J* = 8.8 Hz, 2H), 6.47 (dd, *J* = 15.5, 8.5 Hz, 4H), 5.83 (s, 2H), 2.84 (s, 12H), 1.28 (s, 12H). ^13^C NMR (151 MHz, DMSO), *δ* (ppm): 154.33, 149.69, 148.99, 148.83, 144.64, 144.31, 143.33, 137.66, 135.15, 132.19, 132.14, 132.04, 131.07, 129.87, 128.04, 127.95, 127.62, 126.13, 124.07, 111.31, 111.14, 83.89, 62.09, 40.06, 24.62. HR-MS (m/z): calculated for C_48_H_51_BBrN_3_O_2_ [M-Br]: 712.4069, found: 712.4065.

**Calculation.** The HOMO-LUMO gaps of TPyB and TPy were calculated by the Gaussian 09 program at B3LYP/6-31G(d,p) level, and results were visualized by GaussView 5.0.

**H_2_O_2_ Responsiveness.** A series of H_2_O_2_ of different concentrations (0 – 150 µM) were prepared. Each sample was co-incubated with 20 µM TPyB in DMSO/water mixture (1/9, *V*/*V*) for 30 min at room temperature. The test solutions were transferred to standard quartz cells respectively, and the fluorescence spectra were measured under an excitation wavelength of 480 nm.

H_2_O_2_ responsiveness was further verified by mass measurement. A sample of 5 µM TPyB was co-incubated with 50 µM H_2_O_2_ for 30 min in DMSO/water mixture (1/9, *V*/*V*) at room temperature. The sample was then subjected to freeze-drying treatment. After dissolving with HPLC-grade MeOH, the mass measurement was performed to analyze the structural changes of TPyB before and after the reaction.

**Selectivity of H_2_O_2_ Responsiveness.** Different ROS (H_2_O_2_, HOCl, ·OH, and O_2_^·–^) and RNS (NO_2_^–^ and ONOO^–^), cysteine (Cys), glutathione (GSH), and glucose (Glu) solutions (50 µM) were co-incubated with 20 µM TPyB in DMSO/water mixture (1/9, *V*/*V*) for 30 min at room temperature, respectively. The test solutions were transferred to standard quartz cells respectively, and the emission spectra were measured under an excitation wavelength of 480 nm.

**Algal Culture.** *C. pyre* was cultured in BG11 medium in an illumination incubator under conditions of 25 ºC, a 12 h light/12 h dark cycle, and an illuminance set at 40%. The culture flasks were manually shaken three times daily. The concentration of algae suspension was determined by measuring the optical density at 680 nm (OD_680_).

**CLSM Imaging** **of *C. pyre* Cells.** *C. pyre* cells (2 mL, OD_680_ = 2.0) were incubated with 20 μM TPyB in BG11 for 30 min. After centrifugation to remove the supernatant (7000 rpm, 5 min), the harvested cells were resuspended in 50 μL of PBS. 5 μL of as-prepared sample was placed on a glass slide, covered with a coverslip, and imaged under CLSM using a 100× oil lens. Imaging conditions: *λ*_ex_ = 543 nm and *λ*_em_ = 600-650 nm for TPyB.

**Algal Cell Membrane** **Localization.** Co-staining experiments of *C. pyre* with TPyB (20 μM) and the cell membrane probe DiI (10 μM) were performed according to previous report.^[1]^ Two control groups were included: *C. pyre* stained with TPyB alone and *C. pyre* stained with DiI alone. Imaging conditions: *λ*_ex_ = 543 nm and *λ*_em_ = 580-650 nm for TPyB; *λ*_ex_ = 543 nm and *λ*_em_ = 550–580 nm for DiI.

To further verify the membrane localization of TPyB, we compared the TPyB fluorescence intensity in *C. pyre* with or without cell wall removal after TPyB pre-treatment. Briefly, *C. pyre* suspension (OD_680_ = 2.0) was incubated with 20 μM TPyB in BG11 for 30 min at room temperature. After centrifugated (7000 rpm, 5 min) to remove the unbound TPyB, the remaining cells were resuspended in BG11. 5 mL of the asprepared sample was then treated with a plant protoplasts isolation kit to remove the cell wall, while the other group (without cell wall removal) was subjected to the same procedure without the digestive enzymes solution. 1 mL of each sample was centrifugated (7000 rpm, 5 min) to collect the *C. pyre* cells, and the pellets were resuspended in 50 μL PBS for CLSM imaging. Imaging conditions: *λ*_ex_ = 543 nm and *λ*_em_ = 600-650 nm for TPyB.

**Flow Cytometric Analysis of TPyB Binding to *C. pyre*.** 2 mL of *C. pyre* suspension (OD_680_ = 2.0) was incubated with 20 μM TPyB in BG11 for 30 min at room temperature. Cells were harveted by centrifugation (7000 rpm, 5 min) and resuspended in 500 μL of PBS. The sample was analyzed on a flow cytometer, with the fluorescence signal of approximately 20,000 cells collected in the PE-Texas Red-A channel.

**ITC Analysis of TPyB Binding to *C. pyre*.** A 600 μL of *C. pyre* suspension (OD_680_ = 1.0) or an equal volume of PBS was loaded into the sample cell, and the reference cell was filled with 765 μL of PBS buffer. TPyB solution (150 µM) was sequentially injected into the sample cell in 23 consecutive aliquots of 10 μL each under stirring of 78 rpm. The ITC measurements were independently replicated twice. To eliminate the interference of dilution effects, the dilution enthalpy of TPyB (determined from the titration of TPyB solution into PBS) was subtracted from the raw titration data. The binding thermodynamic parameters of TPyB with the *C. pyre* were obtained by fitting the corrected ITC titration curve.

**SEM Characterization of Algal Morphology.** 2 mL of *C. pyre* suspensions (OD_680_ = 2.0), untreated or treated with 20 µM TPyB for 30 min, were centrifuged to collect the cells. The pellets were washed with PBS, and then resuspended in 50 μL PBS. 10 μL of the samples were placed on a silicon wafer and air-dried. The samples were then fixed with 2.5% glutaraldehyde in PBS solution at 4 ºC for 6 h, followed by three washes with PBS, and then dehydrated in a graded ethanol series (50, 70, 80, 90, 95, and 100%, 15 min for each step). The prepared samples were vacuum‑dried and sputter-coated with platinum for SEM observation.

**Zeta Potential Measurements.** *C. pyre* suspensions (OD_680_ = 2.0), untreated or treated with 20 µM TPyB for 30 min at 25 ºC, were centrifuged (7000 rpm, 5 min) to remove the supernatant. The resulting cell pellets were resuspended in 2 mL of BG11. Zeta potentials of the samples were measured with a DTS1070 disposable capillary cell at 25 ºC on a Nano ZS instrument.

**Growth Curve.** Logarithmic-phase *C. pyre* cells were inoculated into fresh BG11 medium at an initial OD_680_ of about 0.3. Different concentrations of TPyB or TPy were added. The samples were incubated in an illumination incubator, and monitored daily by OD_680_ measurements. All experiments were performed in triplicate.

**Electron Generation.** *C. pyre* cells (2 mL, OD_680_ = 2.0) were pre-incuabted without and with 0.5 μM TPyB for 15 min, followed by adding the artificial electron acceptor DCPIP (100 µM). The absorbance at 600 nm of DCPIP was monitored over irradiation time under white light (900 μmol·m^-2^·s^-1^), UV light (4.6 μmol·m^-2^·s^-1^), blue light (400 μmol m^-2^ s^-1^) or green light (400 μmol m^-2^ s^-1^).

**Chlorophyll Fluorescence Kinetics and Parameter Measurements.** *C. pyre* suspensions (2 mL, OD_680_ = 2.0) were pre-incuabted without and with 0.5 μM TPyB for 30 min and transferred to the sample cell. After 30 min of dark adaptation, the chlorophyll fluorescence kinetics curves and dark-adapted parameters were recorded on a chlorophyll fluorescence monitor. For light-adapted parameters, the samples were illuminated under white light (900 μmol·m^-2^·s^-1^) for 30 min before measurement.

**Oxygen Evolution.** *C. pyre* suspensions (2 mL, OD_680_ = 2.0) were incubated with different concentrations of TPyB in BG11 at 25 ºC for 30 min. Samples were then transferred to the liquid-phase oxygen electrode chamber, and oxygen evolution was recorded at 25 ºC under varying white light intensities of 300-1500 μmol·m^-2^·s^-1^ on a liquid-phase oxygen electrode system.

**ATP Content.** 2 mL of *C. pyre* cells (OD_680_ = 2.0) were pretreated with or without 0.5 µM TPyB for 30 min and then exposed to white light (900 μmol·m^-2^·s^-1^) for 30 min. The cells were harvested by centrifugation (7000 rpm, 5 min), resuspended with 200 μL lysis buffer, and sonicated in an ice bath for 20 min. After centrifugation (10000 rpm, 10 min) at 4 ºC, the supernatant was transferred to a black 96-well plate. The ATP content was then determined using an enhanced ATP assay kit, with the luminescence intensity recorded on a microplate reader.

**NADPH and NADP^+^ Content.** *C. pyre* cells were pretreated following the same procedure used for ATP measurement, and the obtained supernatant was added to a 96-well plate. The content of NADPH and NADP^+^ was then measured using an enhanced NADP^+^/NADPH assay kit, with the absorbance recorded at 450 nm on a microplate reader.

**Rubisco Activity.** *C. pyre* cells (2 mL, OD_680_ = 2.0) were pretreated with or without 0.5 µM TPyB for 30 min and then exposed to white light (900 μmol·m^-2^·s^-1^) for 30 min. The cells were collected via centrifugation (7000 rpm, 5 min), resuspended in 500 μL of extraction buffer, and sonicated for 20 min in an ice bath. After centrifugation (10000 rpm, 10 min) at 4 ºC, the resulting supernatant was mixed with assay buffer. The absorbance of nicotinamide adenine dinucleotide (NADH) at 340 nm was monitored at 30 s intervals using a spectrophotometer, with assay buffer alone as the blank control. Rubisco activity was calculated based on the decrease in NADH absorbance according to the previous report.^[2]^

**Lipid Content Measurement.** *C. pyre* suspensions (initial OD_680_ = 0.3) were incubated with and without 0.5 µM TPyB for 12 days in an illumination incubator. 20 mL of samples were then processed for lipid extraction and content evaluation according to the gravimetric method described in the reference.^[3]^

**CLSM Imaging of** **In situ Reaction of TPyB with H_2_O_2_.** *C. pyre* suspension (2 mL, OD_680_ = 2.0) were incubated with TPyB (20 μM) in BG11 for 30 min. 100 µM H_2_O_2_ was added and incubated for 90 min. Cells were pelleted by centrifugation (7000 rpm, 5 min), resuspended in 50 μL of PBS, and 5 μL of the suspension was placed on a glass slide for imaging. Control groups included *C. pyre* treated with TPyB alone or H_2_O_2_ alone. Imaging conditions: *λ*_ex_ = 543 nm and *λ*_em_ = 600-650 nm for TPyB; *λ*_ex_ = 405 nm and *λ*_em_ = 450-550 nm for TPy.

**In situ Reaction Dynamics of TPyB with H_2_O_2_ by Fluorescence Spectroscopy and Flow Cytometry Analysis.** *C. pyre* suspension (2 mL, OD_680_ = 2.0) were incubated with TPyB (20 μM) in BG11 for 30 min. 100 µM H_2_O_2_ was added and incubated for different times. Fluoresence spectra of samples were then recorded under *λ*_ex_ = 400 nm. For flow cytometry analysis, the cells were collected by centrifugation (7000 rpm, 5 min), and resuspended in 500 μL of PBS. Samples were analyzed on a flow cytometer, with fluorescence signals from approximately 20,000 cells collected in the PE‑Cy5‑A channel.

**Monitoring of** **High Light (HL) Stress.** *C. pyre* suspension (2 mL, OD_680_ = 2.0) in BG11 were first exposed to HL (2000 μmol·m^-2^·s^-1^) for different time (0.5 h or 1 h) at room temperature. TPyB (20 μM) was added to the suspension and incubated for different periods (0.5 h, 1.5 h or 2.5 h). Subsequently, the cells were pelleted by centrifugation (7000 rpm, 5 min), resuspended in 50 μL of PBS, and 5 μL of the final suspension was placed on a glass slide for imaging. *C. pyre* suspension was not exposed to HL but incubated with TPyB for 2.5 h as the control. Imaging conditions: *λ*_ex_ = 543 nm and *λ*_em_ = 600-650 nm for TPyB; *λ*_ex_ = 405 nm and *λ*_em_ = 450-550 nm for TPy.

**Assessment of Intracellular ROS after** **HL Stress by DCFH-DA.** *C. pyre* cells (2 mL, OD_680_ = 2.0) were pretreated with or without 1.0 µM TPyB for 30 min, and then subjected to HL stress (2000 μmol·m^-2^·s^-1^) for 1 h. The samples were transferred to an illumination incubator and allowed to recover for different times (0, 0.5, 1.0 and 1.5 h). DCFH-DA (50 μM) was then added and incuabted for 10 min. Fluorescence spectra of DCFH-DA were recorded at an excitation wavelength of 488 nm.

**Assessment of Intracellular H_2_O_2_.** *C. pyre* cells (2 mL, OD_680_ = 2.0) were pretreated with or without 1.0 µM TPyB for 30 min, followed by 1 h of HL (2000 μmol·m^-2^·s^-1^), and a subsequent 2 h recovery in an illumination incubator. The cells were then harvested by centrifugation (7000 rpm, 5 min), resuspended with 1 mL of acetone, sonicated in an ice-water bath for 30 min. Then the samples were centrifuged (10000 rpm, 10 min) at 4 ºC to collect the supernatant. H_2_O_2_ levels were determined using a H_2_O_2_ assay kit by recording the absorbance at 415 nm with a spectrophotometer.

***F*_v_/*F*_m_ and** ***ϕ*PSII Parameters in *C. pyre* following HL or High Temperature (HT) Stress.** *C. pyre* suspensions (2 mL, OD_680_ = 2.0) were pretreated with or without 1.0 µM TPyB for 30 min, and then subjected to either HL (2000 μmol·m^-2^·s^-1^) or HT (40 ºC) stress for 1 h. The samples were transferred to an illumination incubator and allowed to recover for varying times (0, 1h or 2h). For *F*_v_/*F*_m_ measurement, the as-prepared samples were dark-adapted for 30 min and the *F*_v_/*F*_m_ parameters were recorded on a chlorophyll fluorescence monitor. For *ϕ*PSII measurement, the samples were illuminated under white light (900 μmol·m^-2^·s^-1^) for 30 min before measurement.

**Oxygen Evolution in *C. pyre* after Stress.** *C. pyre* cells were pretreated according to the same procedure used for the *F*_v_/*F*_m_ and *ϕ*PSII parameter measurement. The as-prepared samples were then transferred to the chamber of a liquid-phase oxygen electrode system. Oxygen evolution was measured at 25 ºC under a light intensity of 900 μmol·m^-2^·s^-1^.

**Chlorophyll Content in *C. pyre* after HL Stress.** 2 mL of *C. pyre* cells (OD_680_ = 2.0) were incubated with and without 1.0 μM TPyB for 30 min. The samples were exposed to HL (2000 μmol·m^-2^·s^-1^) stress for 1 h and transferred to an illumination incubator for 2 h of recovery. Then, the cells were harvested by centrifugation (7000 rpm, 5 min), and resuspended in 2 mL of an acetone and ethanol mixture (*V*/*V* = 1:1). After 30 min of extraction in the dark, the mixture was centrifuged (7000 rpm, 5 min). The absorbance of the supernatant was measured at 645 nm (OD_645_) and 663 nm (OD_663_), and the chlorophyll content was calculated using the formula provided in the reference.^[4]^ The experiments were performed in triplicate.

**SOD Activity in *C. pyre* after Stress.** *C. pyre* cells (2 mL, OD_680_ = 2.0) were pretreated with or without 1.0 µM TPyB for 30 min, followed by HL or HT stress for 1 h, and a subsequent 2 h recovery in an illumination incubator. The cells were collected by centrifugation (7000 rpm, 5 min), lysed with 1 mL of lysis buffer, sonicated in an ice bath for 30 min, and then centrifuged at 4 ºC (10000 rpm, 10 min) to collect the supernatant. SOD activity was measured using a WST-8-based total SOD assay kit, with the absorbance at 450 nm recorded on a microplate reader.

**MDA Content in *C. pyre* after Stress.** The MDA content in *C. pyre* cells was determined with a MDA assay kit. Following the same pretreatment used for SOD activity measurement, C. pyre cells were harvested by centrifugation, resuspended in 5 mL of PBS, and sonicated in an ice-water bath for 30 min. 1 mL of the supernatant was reacted with 4 mL of the assay kit reagent at 100 ºC for 15 min. After cooling to room temperature, the samples were centrifuged (3000 rpm, 10 min) to collect the supernatant. MDA content was measured by recording the absorbance at 532 nm of the supernatant on a microplate reader.

**CAT Activity in *C. pyre* after Stress.** Following the same pretreatment used for SOD activity measurement, *C. pyre* cells were harvested by centrifugation, resuspended in 1 mL of lysis buffer, and sonicated in an ice-water bath for 30 min. The supernatant was collected by centrifugation at 4 ºC (10000 rpm, 10 min). 4 µL of the supernatant were transferred to a centrifuge tube and diluted to 40 μL with CAT assay buffer. The reaction was initiated by adding 10 µL of 250 mM H_2_O_2_, incubated at 25  ºC for 2 min, then stopped with 450 μL of catalase stop solution. 10 μL of this mixture was diluted with 40 μL of catalase assay buffer. 10 μL of the diluted sample was transferred to a 96-well plate, mixed with 200 μL of chromogenic working solution, incubated at 25  ºC for 15 min. Absorbance at 520 nm was then measured on a microplate reader.

**Statistical Analysis**

Quantitative data were presented as mean ± standard deviation (SD). Significance difference between two groups was determined with one-way analysis of variance (ANOVA) by Origin 2021 software. *P* < 0.05 was the accepted level of significance.

**Supplementary Figures**

**Figure S1.** Synthetic routes for TPyB.

**Figure S2.** ^1^H NMR spectrum of TPyB in DMSO-*d*_6_.

**Figure S3.** ^13^C NMR spectrum of TPyB in DMSO-*d*_6_.

**Figure S4.** MS spectrum of TPyB.

**Figure S5.** Frontier molecular orbitals (HOMO and LUMO) of TPyB (left) and TPy (right) calculated at the ground state.

**Figure S6.** (a) Relative emission intensity (*I*_max_/*I*_0_) of 20 µM TPyB versus water volume fraction in DMSO/water mixtures. *λ*_ex_ = 480 nm. (b) Size distribution of 20 μM TPyB in DMSO/water mixture with 90% water volume fractions.

**Figure S7.** MS analysis of TPyB before and after H_2_O_2_ treatment.

**Figure S8.** (a) Fluorescence spectra of 20 µM TPy in DMSO/water mixtures with different water volume fractions (*f*_w_, vol%), *λ*_ex_ = 400 nm. (b) Relative emission intensity (*I*_max_/*I*_0_) of TPy versus water fraction in DMSO/water mixtures.

**Figure S9.** CLSM images of *C. pyre* cells (OD_680_ = 2.0) with and without 20 µM TPyB treatment for 30 min. *λ*_ex_ = 543 nm and *λ*_em_ = 600-650 nm.

**Figure S10.** CLSM images of *C. pyre* stained with 20 µM TPyB alone for 30 min (top) or 10 µM DiI alone for 10 min (bottom). *λ*_ex_ = 543 nm and *λ*_em_ = 580-650 nm for TPyB, *λ*_ex_ = 543 nm and *λ*_em_ = 550-580 nm for DiI (false-colored blue).

**Figure S11**. (a) Fluorescence (FL) and bright field (BF) images of TPyB-pretreated *C. pyre* cells with or without cell wall removal. White circles in the BF image indicate the selected cells for fluoresence intensity statistical analysis. *λ*_ex_ = 543 nm and *λ*_em_ = 600-650 nm. (b) Statistical analysis of TPyB fluorescence intensity from 8 selected cells in CLSM images of (a). Error bars: mean ± SD (*n* = 8). *P* ˃ 0.05, ns, not significant.

**Figure S12.** Raw data upon titrating 150 µM TPyB into *C. pyre* suspensions (OD_680_ = 1.0).

**Figure S13.** (a)-(c) Fluorescence spectra of *C. pyre* (OD_680_ = 1.0), 20 µM TPyB and the *C. pyre*/TPyB system under (a) blue light (*λ*_ex_= 480 nm), (b) UV light (*λ*_ex_ = 310 nm) and (c) green light (*λ*_ex_ = 550 nm) excitation.

**Figure S14.** (a)-(d) Absorbance change at 600 nm of DCPIP (100 μM) in the *C. pyre* suspensions without and with 0.5 μM TPyB over irradiation time under (a) white light (900 μmol·m^-2^·s^-1^), (b) blue light (400 µmol·m^-2^·s^-1^), (c) UV (4.6 µmol·m^-2^·s^-1^) and (d) green light (400 µmol·m^-2^·s^-1^). Error bars: mean ± SD (*n* = 3). **P* < 0.05, significant; ***P* < 0.01 and ****P* < 0.001, highly significant.

**Figure S15.** Chlorophyll fluorescence kinetic curves of TPyB, *C. pyre* and *C. pyre*/TPyB.

**Figure S16.** (a) The time course of oxygen evolution of 0.5 μM TPyB under illumination intensity of 900 μmol·m^-2^·s^-1^. (b) Time course of oxygen evolution in *C. pyre* cells treated with different concentrations of TPyB under white light of 900 μmol·m^-2^·s^-1^.

**Figure S17.** (a) Rubisco activity in *C. pyre* without and with 0.5 μM TPyB after 30 min of white light irradiation (900 μmol·m^-2^·s^-1^. (b) Absorbance change at 340 nm of NADH in *C. pyre* suspensions without and with 0.5 μM TPyB over the reaction time. Error bars: mean ± SD (*n* = 3). ***P* < 0.01, highly significant.

**Figure S18**. Oxygen evolution of the *C. pyre*/TPyB biohybrid without or with free TPyB removal under illumination intensity of 900 μmol·m^-2^·s^-1^ over 1500 s.

**Figure S19.** (a) Fluorescence spectra of 20 μM TPyB before and after HL (2000 µmol·m^-2^·s^-1^) exposure for 1 h or 2h. (b) Fluorescence spectra of 20 μM TPyB before and after HT (40 ºC) exposure for 1 h or 2h.

**Figure S20**. CLSM images of *C. pyre* cells (OD_680_ = 2.0) with and without 20 µM TPy treatment for 30 min. *λ*_ex_ = 405 nm and *λ*_em_ = 450-550 nm.

**Figure S21.** (a) Time-dependent fluorescence intensity at 570 nm of the *C. pyre*/TPyB system and *C. pyre* alone after adding 100 µM H_2_O_2_. (b) and (c) Fluorescence spectra of the *C. pyre*/TPyB system and *C. pyre* alone (OD_680_ = 1.0) after the addition of 100 µM H_2_O_2_ for differnet time, respectively. *λ*_ex_ = 400 nm.

**Figure S22.** (a) Flow cytometric analysis of fluorescence distribution change of the *C. pyre*/TPyB system over time after addition of 100 µM H_2_O_2_, with the H_2_O_2_-untreated system as the control. (b) Flow cytometric analysis of the *C. pyre*/TPyB system before and after 2 h reaction with 100 µM H_2_O_2_.

**Figure S23.** Growth curves of *C. pyre* cells in the absence or presence of various concentrations of TPy. Error bars: mean ± SD (*n* = 3).

**Figure S24.** (a) Fluorescence spectra of DCFH-DA (50 μM) in *C. pyre* (OD_680_ = 2.0) followed by 1 h of HL exposure (2000 μmol·m^-2^·s^-1^) and subsequent recovery for different time. (b) Fluorescence spectra of DCFH-DA in the *C. pyre*/TPyB system followed by 1 h of HL exposure (2000 μmol·m^-2^·s^-1^) and subsequent recovery for different time.

**Figure S25.** *F*_v_/*F*_m_ parameters of *C. pyre* without and with 1.0 μM TPyB after exposure to 100 µM H_2_O_2_ for 2 h. Error bars: mean ± SD (*n* = 3). *P* ˃ 0.05, ns, not significant; ^*^*P* < 0.05, significant; ^**^*P* < 0.01, highly significant.

**Figure S26.** (a) Oxygen evolution and (b) time course of oxygen evolution over 1500 s under illumination intensity of 900 μmol·m^-2^·s^-1^ in *C. pyre* cells without or with 1.0 μM TPyB after exposure to 100 µM H_2_O_2_ for 2 h. Error bars: mean ± SD (*n* = 3). ^*^*P* < 0.05, significant; ^**^*P* < 0.01, highly significant.

**Figure S27.** (a) *F*_v_/*F*_m_ and *ϕ*PSII parameters of *C. pyre* without and with treated by 1.0 μM TPy for 30 min. (b) Oxygen evolution of *C. pyre* without or with TPy under illumination intensity of 900 μmol·m^-2^·s^-1^ for 1500 s. Error bars: mean ± SD (*n* = 3). *P* ˃ 0.05, ns, not significant.

**Figure S28.** Oxygen evolution under white light (900 μmol·m^-2^·s^-1^) over 1500 s in *C. pyre* cells and the *C. pyre*/TPyB system after 1 h of HT (40 ºC) exposure and subsequent recovery for different times.

**Figure S29**. Total chlorophyll content in *C. pyre* and *C. pyre*/TPyB biohybrid with or without 1 h of HL (2000 μmol·m^-2^·s^-1^) exposure, followed by 2 h of recovery. Error bars: mean ± SD (*n* = 3). *P* ˃ 0.05, ns, not significant; ^**^*P* < 0.01, highly significant.

**Figure S30.** Relative MDA content in *C. pyre* and *C. pyre*/TPyB system after 1 h of HT (40 ºC) exposure followed by 2 h of recovery.

**Figure S31.** (a) Relative SOD activity and (b) CAT activity in *C. pyre* and *C. pyre*/TPyB system after 1 h of HT (40 ºC) exposure followed by 2 h of recovery.

**Figure S32.** ^1^H NMR spectrum of TPy in DMSO-*d*_6_.

**Figure S33.** ^13^C NMR spectrum of TPy in DMSO-*d*_6_.

**Figure S34.** MS spectrum of TPy.

**References**

[1]	Y. Shen, B. Huang, H. Yao, G. Shan, Y. Shao, X. Zhou, L. Han, C. Zhou, "In-vivo Spectral Recomposition of Sunlight with Glycosylated Aggregation-Induced Emission Antennas for Boosting Photosynthesis " *J. Colloid Interface Sci.* **688**, (2025): 784-795.

[2]	Y. Huang, B. Huang, Y. Shen, Z. Ding, H. Yao, X. Zhou, C. Zhou, L. Han, B. Z. Tang, "Nature-Inspired Artificial Aggregation-Induced Emission Antenna for Assembling with Algae to Promote Photosynthesis" *ACS Appl. Mater. Interfaces* **16**, (2024): 66706-66717.

[3]	V. Matyash, G. Liebisch, T. V. Kurzchalia, A. Shevchenko, D. Schwudke, "Lipid Extraction by Methyl-tert-butyl Ether for High-Throughput Lipidomics " *J. Lipid Res.* **49**, (2008): 1137-1146.

[4]	X. Xu, W. Li, C. Hu, B. Lei, X. Zhang, Y. Li, Q. Zhan, Y. Liu and J. Zhuang, "Promoting the Growth of Mung Bean Plants through Uptake and Light Conversion of NaYF_4_:Yb,Er@CDs Nanocomposites" *ACS Sustainable Chem. Eng.* **8**, (2020): 9751-9762.
